# Supplementary material for: A novel immunopeptidomic-based pipeline for the generation of personalized oncolytic cancer vaccines
Source: eLife. 2022 Mar 22;11:e71156. doi: 10.7554/eLife.71156 (PMC8989416; doi:10.7554/eLife.71156)
Supplement: Supplementary file 1. — For each peptide, the Uniprot ID, gene name, and sequence are reported. Additionally, the last column indicates whether (1) or not (0) the peptide has been already described in a published ligandome dataset. [file elife-71156-supp1.docx]

| **Uniprot ID** | **Gene name** | **Peptide sequence** | **Laumont et al., 2018** |
| --- | --- | --- | --- |
| Q64437 | Adh7 | AGASRIIGI | 1 |
| Q9EQH7 | Ndst3 | FYATIIHDL | 0 |
| O08696 | Foxm1 | SGPNRFILI | 1 |
| Q9CXG9 | Phf19 | QGPEYIERL | 1 |
| Q8R3J5 | Chac1 | KYLNVREAV | 0 |
| Q61001 | Lama5 | HYLPDLHHM | 0 |
| Q09143 | Slc7a1 | SYIIGTSSV | 1 |
| O35495 | Cdk14 | SYIHQRYIL | 1 |
| O08784 | Tcof1 | GYMTPGLTV | 0 |
| Q91ZX7 | Lrp1 | SYLIGRQKI | 1 |
| Q61009 | Scarb1 | RGPYVYREF | 0 |
