# Supplementary material for: A novel immunopeptidomic-based pipeline for the generation of personalized oncolytic cancer vaccines
Source: eLife. 2022 Mar 22;11:e71156. doi: 10.7554/eLife.71156 (PMC8989416; doi:10.7554/eLife.71156)
Supplement: Supplementary file 2. — For each peptide, the Uniprot ID, amino acid sequence, and similar pathogen species with the respective viral peptides with sequence similarity are shown. The last column indicates whether (1) or not (0) the peptide has been already described in a published ligandome dataset. [file elife-71156-supp2.docx]

| **Uniprot ID** | **Peptide sequence** | **Pathogen species** | **Viral Peptide** | **Laumont et al., 2018** | |
| --- | --- | --- | --- | --- | --- |
| **O88738-3** | **SYHPALNAI** | **Molluscum contagiosum virus subtype 1** | **SYHAALNAL** | | **1** |
| **Q9QXZ0** | **AFHSSRTSL** | **Human adenovirus A serotype 31** | **HFSTSRTSL** | | **1** |
| **Q3TWW8** | **SYSDMKRAL** | **Cercopithecine herpesvirus 1** | **AYQDTKRAL** | | **1** |
| **P70452** | **NYNSVNTRM** | **Human herpesvirus 7** | **FYNSVNTRN** | | **0** |
| **Q80TP3** | **SYLTSASSL** | **Influenza A virus** | **TIWTSASSI** | | **0** |
| **Q8VCF0** | **SYLPPGTSL** | **Epstein-Barr virus** | **TYLPPSTSS** | | **1** |
| **O70405** | **FYEKNKTLV** | **Orf virus** | **NYYKNKSLV** | | **0** |
| **Q9D1R1** | **FYKNGRLAV** | **Human adenovirus F serotype 41** | **AYMNGRVAV** | | **0** |
| **Q91XE7** | **KGPNRGVII** | **Variola virus** | **KNPNRFVIF** | | **1** |
| **Q6URW6-2** | **LYKESLSRL** | **Human cytomegalovirus** | **LYLETLSRI** | | **0** |
| **Q9JL70** | **RYLPAPTAL** | **Influenza C virus** | **RNMPAATAL** | | **1** |
| **O54692** | **KYIPAARHL** | **Human cytomegalovirus** | **SHQPAARRL** | | **1** |
| **P54775** | **YYVRILSTI** | **Molluscum contagiosum virus subtype 1** | **YVFRLLSTI** | | **1** |
| **P54775** | **SYRDVIQEL** | **Human cytomegalovirus** | **RYADVIQEV** | | **0** |
| **Q61036** | **KFYDSKETV** | **Human adenovirus A serotype 18** | **NFYNSKETV** | | **1** |
