# Supplementary figures and images for: A novel immunopeptidomic-based pipeline for the generation of personalized oncolytic cancer vaccines
Source: eLife. 2022 Mar 22;11:e71156. doi: 10.7554/eLife.71156 (PMC8989416; doi:10.7554/eLife.71156)

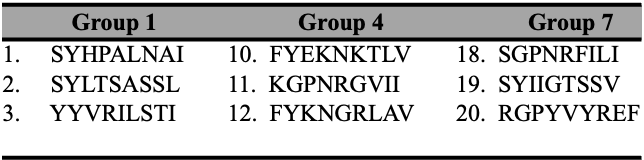

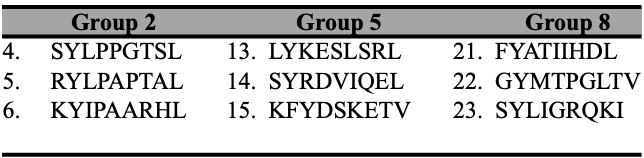

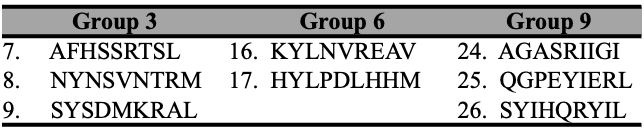

Supplement: Supplementary file 3. — For each group of mice, the peptides with the respective identification number as indicated in the ELISpot assay are reported. [file elife-71156-supp3.docx]
