# Supplementary material for: A novel immunopeptidomic-based pipeline for the generation of personalized oncolytic cancer vaccines
Source: eLife. 2022 Mar 22;11:e71156. doi: 10.7554/eLife.71156 (PMC8989416; doi:10.7554/eLife.71156)
Supplement: Supplementary file 4. — The candidate peptides used in PeptiCRAd technology with the respective net charge without and with the poly-lysine modification are shown. [file elife-71156-supp4.docx]

| **Name of peptide** | **Peptide sequence** | **Net charge pH 7** | | **Poly-lysine peptide** | **Net charge pH 7** | |
| --- | --- | --- | --- | --- | --- | --- |
| **Peptide 1** | **SYLPPGTSL** | **0** | **KKKKKKSYLPPGTSL** | | | **6** |
| **Ppetide 2** | **RYLPAPTAL** | **1** | **KKKKKKRYLPAPTAL** | | | **7** |
| **Peptide 3** | **KYIPAARHL** | **2.1** | **KKKKKKYIPAARHL** | | | **7.1** |
| **Peptide 4** | **LYKESLSRL** | **1** | **KKKKKKLYKESLSRL** | | | **7** |
| **Peptide 5** | **KYLNVREAV** | **1** | **KKKKKKYLNVREAV** | | | **6** |
| **Peptide 6** | **FYATIIHDL** | **-0.9** | **KKKKKKKFYATIIHDL** | | | **6.1** |
| **Peptide 7** | **SPSYAYHQF** | **0.1** | **KKKKKKSPSYAYHQF** | | | **6.1** |
