# Supplementary material for: A novel immunopeptidomic-based pipeline for the generation of personalized oncolytic cancer vaccines
Source: eLife. 2022 Mar 22;11:e71156. doi: 10.7554/eLife.71156 (PMC8989416; doi:10.7554/eLife.71156)
Supplement: Supplementary file 5. — The poly-lysine-modified peptides, Uniprot ID, and respective gene names for each PeptiCRAd treatment group are summarized. [file elife-71156-supp5.docx]

| **Peptide** | **Uniprot ID** | **Gene name** |  |
| --- | --- | --- | --- |
| **KKKKKKSYLPPGTSL** | **Q8VCF0** | **MAVS** | **PeptiCRAd1 (PC1)** |
| **KKKKKKRYLPAPTAL** | **Q9JL70** | **FANCA** |  |
| **KKKKKKYIPAARHL** | **O54692** | **ZW10** | **PeptiCRAd2 (PC2)** |
| **KKKKKKLYKESLSRL** | **Q6URW6-2** | **MYH14** |  |
| **KKKKKKYLNVREAV** | **Q8R3J5** | **Chac1** | **PeptiCRAd3 (PC3)** |
| **KKKKKKKFYATIIHDL** | **Q9EQH7** | **Ndst3** |  |
